# Associations between Red Blood Cell Transfusions and Necrotizing Enterocolitis in Very Low Birth Weight Infants: Ten-Year Data of a Tertiary Neonatal Unit

**DOI:** 10.3390/medicina55010016

**Published:** 2019-01-15

**Authors:** Justinas Teišerskas, Rūta Bartašienė, Rasa Tamelienė

**Affiliations:** 1 King’s College Hospital NHS Foundation Trust, Princess Royal University Hospital, Farnborough Common, Orpington, Kent BR6 8ND, UK; 2 Department of Obstetrics and Gynaecology, Hospital of Lithuanian University of Health Sciences Kauno klinikos, LT-50161 Kaunas, Lithuania; ruta.damosiute@gmail.com; 3 Department of Neonatology, Faculty of Medicine, Medical Academy, Lithuanian University of Health Sciences, LT-44307 Kaunas, Lithuania; rasa.tameliene@kaunoklinikos.lt

**Keywords:** blood transfusion, necrotizing enterocolitis, neonate, premature infants, very-low-birth-weight infant

## Abstract

*Background and Objective*: Necrotizing enterocolitis (NEC) remains an important cause of mortality in preterm neonates. There are many risk factors for NEC; however, probably the most controversial one is red blood cell transfusions (RBCT). The data concerning the link between NEC and RBCT has been conflicting. Therefore, we aimed to analyze the association between NEC and RBCT in Neonatal Intensive Care Unit (NICU) at the Hospital of Lithuanian University of Health Sciences. *Materials and Methods*: We used the Very Low Birth Weight (VLBW) Infants database to match all infants with ≥2a Bell’s stage NEC admitted between 1 January 2005 and 31 December 2014 (*n* = 54) with a control group (*n* = 54) of similar gestational age and birth weight and without NEC. We analyzed the charts of these infants and performed statistical analysis on 20 clinical variables including RBCT. *Results*: The main clinical and demographic characteristics did not differ between the two groups. All variables associated with RBCT (receipt of any RBCT, the number of transfusions and the volume transfused in total) were significantly higher in the NEC group both before the onset of NEC and throughout the hospitalization. RBCT increased the odds of NEC even after adjustment for confounding factors. In addition, we found that congenital infection was more abundant in the NEC group and increased the odds of NEC 2.7 times (95% confidence interval CI (1.1, 6.3), *p* = 0.024). *Conclusions*: A higher number and the total volume of RBCT are associated with an increased risk of NEC in VLBW infants. The presence of congenital infection might identify the infants at risk.

## 1. Introduction

Necrotising enterocolitis (NEC) is a serious inflammation of the bowel which mostly affects preterm infants [1]. Two to five percent of all admissions to the Neonatal Intensive Care Unit (NICU) are because of NEC [2]. In 85% cases, NEC affects very low birth weight (VLBW) infants (<1500 g) [3] and is a leading cause of mortality and morbidity in preterm neonates [1]. The mortality rate of NEC in general is around 30%, while extremely low birth weight infants (<1000 g) and those who need surgery, have a mortality rate of 40 to 45% [4].

Prematurity is the most important risk factor for NEC because of immature bowel protective barriers and local host defenses, such as secretory immunoglobulin A (IgA), mucosal enzymes, and immature bowel motility [2]. In addition, preterm neonates are more likely to have an abnormal pattern of intestinal colonization (dysbiosis) early at birth, which is perfect for pathogenic bacteria to consolidate and NEC to develop [5]. There are many other risk factors of NEC described in the literature, such as early enteral feedings, [6] absent/reverse end-diastolic umbilical artery Doppler flow, patent ductus arteriosus (PDA) and its treatment with indomethacin or ibuprofen, [1] etc. Hypoxic–ischemic events, such as low Apgar scores, umbilical artery catheterization, episodes of apnea or bradycardia, do not play the main role in the pathogenesis of NEC but may be important additional factors [7,8].

Probably the most controversial risk factor for NEC is red blood cell transfusions (RBCT). The association between NEC and RBCT has been shown in several studies [9,10,11,12,13], but in those studies, infants who were suspected to have a transfusion associated NEC were more likely to be premature, had lower birth weight or had higher unadjusted mortality rate [14]. Because of that, many discussions arose, and some of the more recently published studies questioned this association [15,16]. Our study aimed to analyze the association between NEC and RBCT in the Hospital of Lithuanian University of Health Sciences Kauno Klinikos (LUHSH KK) NICU.

## 2. Materials and Methods

This is a retrospective case-control study. We used the Very Low Birth Weight Infants database established in our NICU in 1991 to identify all infants diagnosed with second or higher greater stage NEC according to modified Bell‘s criteria [17] who were hospitalized to the NICU of LUHSH KK from 1 January 2005 until 31 December 2014. These infants were defined as the NEC case group (*n* = 54). Our control group (*n* = 54) was selected by matching each NEC group patient with an infant born after the NEC group patient who had a similar gestational age and birth weight and had no diagnosis or even suspicion of NEC.

In both the control and case group, there were no patients who would have been deemed to be excluded from the study (e.g., born with congenital anomalies, term babies, spontaneous intestinal perforation within 48 h of life, etc.) Probiotics were not used during the period of this study and, since there was no donor milk bank operation in place, all newborns were given artificial term formula in place of lacking maternal milk.

After identifying the patients, we retrieved their clinical records and analyzed them. We defined RBC transfusions before NEC diagnosis as those performed before the first symptoms of NEC occurred (the mean day of first NEC symptoms in the NEC group was day 14 after birth). Therefore, the frequency and volume of transfusions were counted until 14 days after birth in the control group.

We recorded patient history, including gestational age, birth weight, Apgar scores at one and five minutes, the presence of respiratory distress syndrome (RDS), whether surfactant was used, the predominant type of enteral feeds at the time of NEC occurrence (day 14 in control group), the presence of PDA and whether ibuprofen was administered. PDA was diagnosed based on echocardiographic findings. The presence of congenital infection was confirmed when at least two clinical or biochemical findings suggestive of septicemia were present within 48 h after birth and at least 5-day course of antibiotics was completed.

The decision for RBCT was made by neonatologist caring for the infant using the transfusion guidelines (Table 1) and packed red blood cells (PRBC) volume in milliliters needed to transfuse was calculated using formula weight(kg) × 80 mL/kg × (Ht(desired)–Ht(current)) × Ht (PRBC)^−1^. Enteral feeding was withheld only for the duration of transfusion.

The number of transfused patients, the frequency of transfusions per patient and the amount of transfused PRBC (ml) was compared in NEC and control groups. The Statistical analysis was performed with SPSS ver. 17.0. The normal distribution of data was tested using Kolmogorov–Smirnov test. Normally distributed numerical data were expressed as mean (SD), and *t*-test was used to determine statistically significant differences, whereas non-normally distributed data were expressed as median (IQR) and Mann–Whitney test was used. Chi-square test was used for categorical data. In logistic regression analysis, the NEC outcome was coded as 1 and the absence of NEC as 0. In multivariate analysis logistic regression model was adjusted for possible risk and protective factors for NEC: gestational age, Apgar score at 1 min, congenital infection, PDA, ibuprofen, RDS and surfactant. *p* value under 0.05 was considered significant.

All subjects’ parents/legal guardians gave their signed consent for using depersonalized medical record data for research purposes upon admission to the hospital. The study was conducted in accordance with the Declaration of Helsinki, and the protocol was approved by Kaunas Regional Biomedical Ethics Committee on 8 October 2018 (Approval no. BE-2-79).

## 3. Results

Out of all VLBW infants admitted to the NICU from January 2005 to December 2014, a total of 108 infants (54 cases and 54 matched controls) were included in the study. The mean age at onset of NEC was 14 days (range 3–49). Thirty-four patients (63%) were classified as NEC stage 3b, six (11%) as type 3a, five (9%) as 2b, and nine (17%) patients as stage 2a. Forty patients (74.1%) were operated by laparotomy. Out of 32 cases who developed NEC after transfusion, 25% (8) infants showed first symptoms within 24 h and a total of 50% (16) infants within 48 h from the last transfusion.

NEC cases and controls had similar mean gestational age, birth weight, maternal age, and Apgar scores (Table 2). The characteristics reaching significance in univariate analysis were congenital infection, death and all those related to transfusions (number of patients, number of transfusions in a patient, the volume of PRBC) (Table 2).

Univariate logistic regression analysis showed that NEC cases were more likely to be transfused than controls before NEC diagnosis. Furthermore, the risk of NEC increased 1.5 times with a higher number of transfusions before NEC, and each mL of PRBC transfused was associated with a 3.8% increase in the odds of NEC. Congenital infection itself increased the odds of NEC 2.7 times (Table 3).

Multivariate logistic regression model included these covariates: gestational age, Apgar score at one minute, congenital infection, PDA, RDS and use of ibuprofen and surfactant. This analysis showed similar results with even higher odds for NEC with each predictor (Table 3).

## 4. Discussion

NEC remains a leading cause for morbidity and mortality in preterm infants [1] with a reported incidence of around 11% among VLBW infants [18]. In our study, the incidence of NEC was 6.2% over the ten-year period with annual variations ranging from 2.3% to 11.9% (data taken from the VLBW infants’ database). The timing of NEC in this study (mean chronological age at onset 14 days, range 3 to 49 days) was also similar to the reported peak of NEC at nine to 38 days of life (range two to 56 days) [19], thus, minimizing the possibility of including spontaneous intestinal perforations, which usually occur before day seven of life. Most of the cases reported in this study were of definite/severe NEC since the majority of them (63%) were classified as modified Bell’s stage 3b, and 74.1% of all cases were treated surgically. With regard to the definition of transfusion-related gut injury, in our study, 50% of infants (*n* = 32) developed NEC within 48 h of transfusion, which falls into the range of 34.7% to 77.8% reported in other studies [19,20,21].

When considering possible risk factors, we did not find an association between NEC and gestational age, birth weight, sex, Apgar scores at one and five minutes of life, RDS (plus surfactant) or PDA (plus ibuprofen), which was consistent with the similar study of Martin et al. [22]. On the other hand, in a study, which had a sample three times bigger than ours (111 cases and 222 controls) PDA and treatment with indomethacin were found to differ significantly between cases and controls [21]. Moreover, assuming that NEC can be caused by ischemic events early in life [1], low Apgar scores were found to be contributing to the NEC cases in Bak et al., but the number of cases in that study was only 18 and the control group was not matched to the case group [20]; furthermore, bigger studies did not confirm this fact [19,21,22].

A strong correlation between NEC and congenital infection has been found in this work: Significantly more infants in the NEC group had a congenital infection (79.6% vs. 59.3%, *p* = 0.022) and its calculated odds-ratio for NEC was 2.7 (95% 1.1, 6.3, *p* = 0.024). We could not find any studies in which a variable of congenital infection was analyzed, however, other infection characteristics, such as sepsis [19], chorioamnionitis [21], or nosocomial sepsis [22], were previously found to be of no significance between NEC and control groups. Thus, it is possible that the diagnosis of congenital infection could be another unique predictor of NEC in VLBW infants, especially considering the fact that antibiotic therapy did not modify the occurrence of NEC in our study (92.6% of infants in both groups received antibiotics before NEC, *p* = 1.000).

We found that all three variables describing transfusion before NEC correlated with NEC compared very similarly to some previous studies. Transfusion as a nominal variable had an adjusted odds ratio (OR) of 3.0 (95% CI 1.1, 8.0, *p* = 0.034), which is almost identical to that reported by Wan-Huen et al. [19] For transfusion as an ordinal variable, the unadjusted OR was 1.5 (95% CI 1.0, 2.2, *p* = 0.04), which is the same as in Martin et al. [22] and the adjusted OR (1.6, 95% CI 1.0, 2,5, *p* = 0.056), although of borderline statistical significance, is comparable to the report of Bak et al. [20] Moreover, in our study the volume of PRBC transfused also significantly correlated with increased odds of NEC (adjusted OR 1.04, 95% CI 1.0, 1.1, *p* = 0.043).

In terms of outcomes, more infants in the NEC group (47 vs. 27, *p* < 0.001) required more transfusions (three vs. 0.5, *p* < 0.001) during hospital course and significantly, more infants died in the NEC group (20 vs. three, *p* < 0.003). All results are similar to previously reported data [19,21].

Although we found an association of PRBC transfusion and NEC, it does not infer a causal relationship. Since the signs of anemia and early NEC can be similar and are non-specific (pallor, apnea, tachycardia) [15], it is difficult to differentiate between these two conditions. In this study, we tried to minimize these confounders by carefully reviewing the clinical course of NEC case-by-case in medical records and assignment.

Unfortunately, due to maternal medical records being unavailable, we could not report the rate of administration of antenatal steroids, which was shown to be protective against NEC in gestational ages >25 weeks [23]. In addition, true culture-positive sepsis has not been separated in this analysis from fetal inflammatory response syndrome while reporting and accounting for the exposure to antibiotics. 

Moreover, in a recent prospective multicenter study of 600 infants, it was found that severe anemia and not PRBC transfusion increased the risk of NEC [24]. Therefore, since our investigation did not include variables of hemoglobin level, hematocrit or vital signs, we could not have discriminated between infants having symptoms of anemia or NEC. On the other hand, a recent high-powered propensity-matched study confirmed that surgical NEC occurs at higher rates after PRBC transfusion [25].

An ongoing multi-center, randomized point of care trial titled WHEAT (Withholding Enteral feeds Around packed red cell Transfusion) is aiming to find out whether withholding milk feeds before, during, and after blood transfusion in preterm infants reduces the risk of necrotizing enterocolitis [26]. Therefore, the question of the causal relationship between NEC and PRBC transfusion is yet to be answered.

## 5. Conclusions

Every milliliter of RBC transfused is associated with an increased risk of NEC in VLBW infants. We could not find a correlation with common speculated variables and NEC. However, the presence of congenital infection might identify the infants at risk.

## Figures and Tables

**Table 1:** Packed red blood cells (PRBC) transfusion guidelines at Kaunas Clinics Neonatal Intensive Care Unit (NICU) valid at the time of the study.

Hb ≤ 110 g/l Ht ≤ 0.35	Infants requiring mechanical ventilation with MAP > 6–8 cm H_2_O and FiO_2_ > 40–50%
Hb ≤ 100 g/l Ht ≤ 0.30	Infants requiring minimal mechanical ventilation or CPAP > 6 cm H_2_O and FiO_2_ ≤ 40% Before surgery (according to the extent of surgery)
Hb ≤ 80 g/l Ht ≤ 0.25	One or more criteria present: tachycardia (heart rate ≥180 beats per minute) for ≥24 h and/or tachypnea (respiratory rate ≥80 breaths per minute) for ≥24 h, without an alternative cause for these symptoms increase of oxygen requirement from the previous 48 h or increase of CPAP by 20% (e.g., from 10 cm H_2_O to 12 cm H_2_O) weight gain <10 g/kg per day over the previous four days while receiving >100–120 kcal/kg per day Apnea with bradycardia requiring positive-pressure ventilation despite adequate doses of xanthines: >1 episode per hour or >2 episodes per 24 h Before surgery (according to the extent of surgery)
Hb ≤ 70 g/l Ht ≤ 0.20	Asymptomatic infants with an absolute reticulocyte count <100,000/μL (<2 percent)

CPAP—continuous positive airway pressure; FiO_2_—fraction of inspired oxygen; Hb—hemoglobin; Ht—hematocrit; MAP—mean airway pressure.

**Table 2:** Demographic and clinical characteristics of the cases and controls.

Characteristic	NEC Cases *n* = 54	Controls *N* = 54	*p*-Value *
Gestational age in weeks, mean (SD)	26.5 (2.4)	26.6 (2.0)	0.896
Birth weight in grams, mean (SD)	954 (274)	984 (273)	0.564
Maternal age in years, mean (SD)	26.7 (6.9)	28.9 (6.4)	0.099
Apgar score 1 min, mean (SD)	5 (2)	6 (2)	0.636
Apgar score 5 min, mean (SD)	7 (2)	7 (2)	0.650
Female, *n* (%)	21 (38.9)	29 (53.7)	0.123
PDA, *n* (%)	28 (51.9)	32 (59.3)	0.439
Ibuprofen, *n* (%)	17 (31.5)	20 (37.0)	0.543
RDS, *n* (%)	46 (85.2)	46 (85.2)	1.000
Surfactant, *n* (%)	41 (75.9)	33 (61.1)	0.097
Congenital infection, *n* (%)	43 (79.6)	32 (59.3)	0.022
Predominantly fed breast milk (>80% daily feeds), *n* (%)	36 (66.6)	26 (48.1)	0.051
Antibiotics prior to NEC, *n* (%)	50 (92.6)	50 (92.6) **	1.000
Number of infants transfused prior to NEC, *n* (%)	30 (56.6)	19 (35.2) ^†^	0.026
Number of infants transfused during hospitalization, *n* (%)	47 (87.0)	27 (50.0)	<0.001
Number of PRBC transfusions prior to NEC, median (IQR)	1 (0–3)	0 (0–1) ^†^	0.019 ^‡^
Total number of PRBC transfusions, median (IQR)	3 (0–6)	1 (0–3)	<0.001 ^‡^
Volume of PRBC transfused prior to NEC in mL, mean (SD)	13.4 (15.2)	7.0 (11.1) ^†^	0.015
Total volume of PRBC in mL, mean (SD)	57.8 (46.0)	27.0 (50.0)	0.001
Length of stay in days, if discharged home, mean (SD)	90.0 (47.0)	75.0 (35.1)	0.099
Death, *n* (%)	20 (37.7)	3 (5.7)	<0.001

* *p*-values for continuous variables obtained from *t*-tests and for categorical variables from chi-square analysis with one degree of freedom, if not otherwise noted; ** antibiotics prior to average time of NEC diagnosis (day 14); ^†^ transfusions before mean NEC onset day in case group (day 14); ^‡^ *p*-values obtained from Mann–Whitney test. NEC—necrotizing enterocolitis. PDA—patent ductus arteriosus. PRBC—packed red blood cell transfusion. RDS—respiratory distress syndrome.

**Table 3:** Univariate and multivariate logistic regression analysis of necrotizing enterocolitis (NEC) cases.

	Univariate Logistic Regression			Multivariate Logistic Regression		
Parameter	OR	95% CI	*p*-value	Adjusted * OR	95% CI	*p*-value
Transfusion prior to NEC	2.4	(1.1, 5.2)	0.027	3.0	(1.1, 8.0)	0.034
Number of transfusions prior to NEC	1.5	(1.0, 2.2)	0.04	1.6	(1.0, 2.5)	0.056
Volume of PRBC transfused prior to NEC in mL	1.038	(1.0, 1.1)	0.019	1.040	(1.0, 1.1)	0.043
Congenital infection	2.7	(1.1, 6.3)	0.024	NA	NA	NA

* Conditional logistic regression models adjusted for gestational age, Apgar score at 1 min, congenital infection, PDA, ibuprofen, RDS, and surfactant. NA—not applicable. NEC—necrotizing enterocolitis. PRBC—packed red blood cell transfusion.
